# 7′-Phenyl-5′,6′,7′,7a′-tetra­hydro­dipiro[indan-2,5′-pyrrolo­[1,2-*c*][1,3]thia­zole-6′,2′′-indan]-1,3,1′′-trione

**DOI:** 10.1107/S1600536811046174

**Published:** 2011-11-05

**Authors:** Ang Chee Wei, Mohamed Ashraf Ali, Tan Soo Choon, Ching Kheng Quah, Hoong-Kun Fun

**Affiliations:** a Institute for Research in Molecular Medicine, Universiti Sains Malaysia, 11800 USM, Penang, Malaysia; b X-ray Crystallography Unit, School of Physics, Universiti Sains Malaysia, 11800 USM, Penang, Malaysia

## Abstract

The asymmetric unit of the title compound, C_28_H_21_NO_3_S, contains two mol­ecules with similar geometries. The thia­zolidine rings adopt half-chair conformations while the pyrrolidine and the diketo-substituted five-membered carbocyclic rings are in envelope conformations with the spiro C atoms at the flaps. In one mol­ecule, the phenyl ring forms dihedral angles of 57.76 (12) and 71.79 (12)° with the fused benzene rings and the fused benzene rings form a dihedral angle of 57.75 (13)°. The corresponding dihedral angles in the other mol­ecule are 60.04 (12), 72.93 (12) and 54.51 (13)°. The mol­ecular structure is stabilized by intra­molecular C—H⋯O hydrogen bonds, which generate *S*(6) ring motifs. In the crystal, mol­ecules are linked *via* C—H⋯O and C—H⋯N hydrogen bonds into layers lying parallel to the *ab* plane.

## Related literature

For related structures and background references, see: Wei *et al.* (2011*a* ▶,*b* ▶,*c* ▶); Kumar *et al.* (2010 ▶). For hydrogen-bond motifs, see: Bernstein *et al.* (1995 ▶). For ring conformations, see: Cremer & Pople (1975 ▶). For the stability of the temperature controller used in the data collection, see: Cosier & Glazer (1986 ▶). For bond-length data, see: Allen *et al.* (1987 ▶).
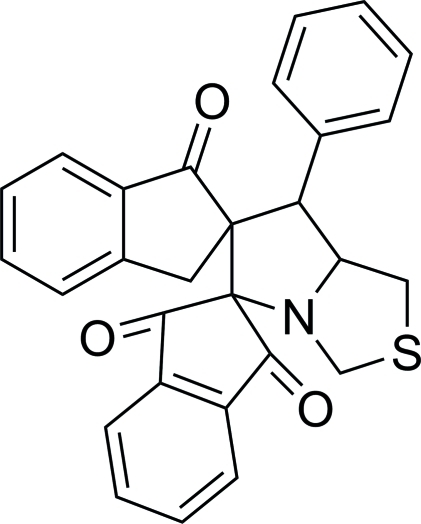


## Experimental

### 

#### Crystal data

C_28_H_21_NO_3_S
*M* *_r_* = 451.52
Monoclinic, 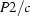

*a* = 19.3315 (6) Å
*b* = 9.6523 (3) Å
*c* = 29.9731 (8) Å
β = 127.817 (2)°
*V* = 4418.1 (2) Å^3^
*Z* = 8
Mo *K*α radiation
μ = 0.18 mm^−1^
*T* = 100 K
0.44 × 0.22 × 0.16 mm

#### Data collection

Bruker SMART APEXII DUO CCD area-detector diffractometer
Absorption correction: multi-scan (*SADABS*; Bruker, 2009 ▶) *T*_min_ = 0.925, *T*_max_ = 0.973
47347 measured reflections
12947 independent reflections
7530 reflections with *I* > 2σ(*I*)
*R*_int_ = 0.067

#### Refinement

*R*[*F* ^2^ > 2σ(*F* ^2^)] = 0.066
*wR*(*F* ^2^) = 0.155
*S* = 1.02
12947 reflections
595 parameters
H-atom parameters constrained
Δρ_max_ = 0.45 e Å^−3^
Δρ_min_ = −0.49 e Å^−3^

### 

Data collection: *APEX2* (Bruker, 2009 ▶); cell refinement: *SAINT* (Bruker, 2009 ▶); data reduction: *SAINT*; program(s) used to solve structure: *SHELXTL* (Sheldrick, 2008 ▶); program(s) used to refine structure: *SHELXTL*; molecular graphics: *SHELXTL*; software used to prepare material for publication: *SHELXTL* and *PLATON* (Spek, 2009 ▶).

## Supplementary Material

Crystal structure: contains datablock(s) global, I. DOI: 10.1107/S1600536811046174/hb6489sup1.cif

Structure factors: contains datablock(s) I. DOI: 10.1107/S1600536811046174/hb6489Isup2.hkl

Additional supplementary materials:  crystallographic information; 3D view; checkCIF report

## Figures and Tables

**Table 1:** Hydrogen-bond geometry (Å, °)

*D*—H⋯*A*	*D*—H	H⋯*A*	*D*⋯*A*	*D*—H⋯*A*
C15*A*—H15*A*⋯O2*A*^i^	0.95	2.51	3.235 (3)	134
C16*B*—H16*B*⋯N1*B*^ii^	0.95	2.48	3.383 (3)	159
C18*B*—H18*B*⋯O2*B*	0.99	2.33	3.070 (4)	131
C18*A*—H18*C*⋯O2*A*	0.99	2.40	3.167 (4)	133
C19*A*—H19*A*⋯O1*A*	1.00	2.37	3.075 (4)	127
C19*B*—H19*B*⋯O1*B*	1.00	2.40	3.086 (4)	125
C23*A*—H23*A*⋯O1*B*^iii^	0.95	2.50	3.209 (4)	132
C23*B*—H23*B*⋯O1*A*	0.95	2.58	3.227 (4)	126

Symmetry codes: (i) 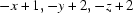; (ii) 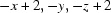; (iii) 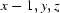.

**Fig. 1.**
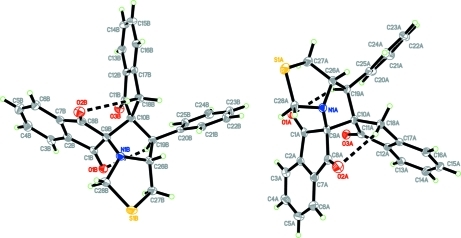
The molecular structure of the title compound showing 50% probability displacement ellipsoids for non-H atoms. Intramolecular hydrogen bonds are shown as dashed lines.

**Fig. 2.**
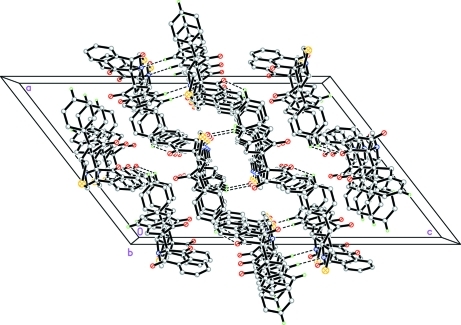
The crystal structure of the title compound, viewed along the b axis. H atoms not involved in hydrogen bonds (dashed lines) have been omitted for clarity.

## supplementary crystallographic information

### Comment

As part of our ongoing search for novel heterocyclic compounds with antitubercular activity (Wei *et al.*, 2011*a*,b,c), our group has synthesized the title compound as described below.

The asymmetric unit (Fig. 1) of the title compound consists of two independent molecules (*A* and *B*), with comparable geometries. The thiazolidine (S1/N1/C26-C28) rings are twisted about S1-C27 bonds, with puckering parameters (Cremer & Pople,1975) Q = 0.423 (2) Å and φ = 22.4 (3)°; and Q = 0.443 (2) Å and φ = 203.1 (3)°, in molecules *A* and *B*, respectively, thereby adopting a half-chair conformation. The pyrrolidine (N1/C9/C10/C19/C27, puckering parameters Q = 0.489 (3) Å and φ = 245.0 (3)° with atom C10A at the flap; and Q = 0.485 (3) Å and φ = 65.8 (3)° with atom C10B at the flap, in molecules *A* and *B*, respectively) and five-membered carbocyclic (C1/C2/C7-C9, puckering parameters Q = 0.176 (3) Å and φ = 138.9 (9)° with atom C9A at the flap; and Q = 0.153 (3) Å and φ = 319.9 (10)° with atom C9B at the flap, in molecules *A* and *B*, respectively) rings are in envelope conformations. Bond lengths (Allen *et al.*, 1987) and angles are within normal ranges and are comparable to the related structures (Kumar *et al.*, 2010; Wei *et al.*, 2011*a*,b,c). In molecule *A*, the phenyl (C20A-C25A) ring forms dihedral angles of 57.76 (12) and 71.79 (12)° with the two benzene (C2A-C7A and C12A-C17A) rings, respectively and the two benzene rings form a dihedral angle of 57.75 (13)°. The corresponding dihedral angles in molecule *B* are 60.04 (12), 72.93 (12) and 54.51 (13)°. The molecular structure is stabilized by intramolecular C18A–H18C···O2A, C18B–H18B···O2B, C19A–H19A···O1A and C19B–H19B···O1B hydrogen bonds (Table 1), which generate *S*(6) ring motifs (Fig. 1, Bernstein *et al.*, 1995).

In the crystal, Fig. 2, molecules are linked *via* intermolecular C15A–H15A···O2A, C16B–H16B···N1B, C23A–H23A···O1B and C23B–H23B···O1A hydrogen bonds (Table 1) into two-dimensional layers parallel to the *ab*-plane.

### Experimental

A mixture of (*Z*)-(2-benzylidene)-2,3-dihydro-1*H*-indene-1-one (0.001 mol), ninhydrin (0.001 mol) and thiazolidine-4-carboxylic acid (0.002 mol) (1:1:2) was dissolved in methanol (10 ml) and refluxed for 4 h. After completion of the reaction as evident from TLC, the mixture was poured into crushed ice. The precipitated solid was filtered, washed and recrystallized from a petroleum ether–ethyl acetate mixture (1:1) to afford the title compound as yellow blocks.

### Refinement

All H atoms were positioned geometrically and refined using a riding model with C–H = 0.95-1.00 Å and *U*_iso_(H) = 1.2 *U*_eq_(C).

### Crystal data

**Table:**

C_28_H_21_NO_3_S	*F*(000) = 1888
*M**_r_* = 451.52	*D*_x_ = 1.358 Mg m^−^^3^
Monoclinic, *P*2/*c*	Mo *K*α radiation, λ = 0.71073 Å
Hall symbol: -P 2yc	Cell parameters from 8142 reflections
*a* = 19.3315 (6) Å	θ = 2.3–29.7°
*b* = 9.6523 (3) Å	µ = 0.18 mm^−^^1^
*c* = 29.9731 (8) Å	*T* = 100 K
β = 127.817 (2)°	Block, yellow
*V* = 4418.1 (2) Å^3^	0.44 × 0.22 × 0.16 mm
*Z* = 8	

### Data collection

**Table:**

Bruker SMART APEXII DUO CCD area-detector diffractometer	12947 independent reflections
Radiation source: fine-focus sealed tube	7530 reflections with *I* > 2σ(*I*)
graphite	*R*_int_ = 0.067
φ and ω scans	θ_max_ = 30.1°, θ_min_ = 1.3°
Absorption correction: multi-scan (*SADABS*; Bruker, 2009)	*h* = −27→27
*T*_min_ = 0.925, *T*_max_ = 0.973	*k* = −13→13
47347 measured reflections	*l* = −41→42

### Refinement

**Table:**

Refinement on *F*^2^	Primary atom site location: structure-invariant direct methods
Least-squares matrix: full	Secondary atom site location: difference Fourier map
*R*[*F*^2^ > 2σ(*F*^2^)] = 0.066	Hydrogen site location: inferred from neighbouring sites
*wR*(*F*^2^) = 0.155	H-atom parameters constrained
*S* = 1.02	*w* = 1/[σ^2^(*F*_o_^2^) + (0.0595*P*)^2^ + 1.4465*P*] where *P* = (*F*_o_^2^ + 2*F*_c_^2^)/3
12947 reflections	(Δ/σ)_max_ = 0.001
595 parameters	Δρ_max_ = 0.45 e Å^−^^3^
0 restraints	Δρ_min_ = −0.49 e Å^−^^3^

### Special details

**Table:**

Experimental. The crystal was placed in the cold stream of an Oxford Cryosystems Cobra open-flow nitrogen cryostat (Cosier & Glazer, 1986) operating at 100.0 (1) K.
Geometry. All esds (except the esd in the dihedral angle between two l.s. planes) are estimated using the full covariance matrix. The cell esds are taken into account individually in the estimation of esds in distances, angles and torsion angles; correlations between esds in cell parameters are only used when they are defined by crystal symmetry. An approximate (isotropic) treatment of cell esds is used for estimating esds involving l.s. planes.
Refinement. Refinement of F^2^ against ALL reflections. The weighted R-factor wR and goodness of fit S are based on F^2^, conventional R-factors R are based on F, with F set to zero for negative F^2^. The threshold expression of F^2^ > 2sigma(F^2^) is used only for calculating R-factors(gt) etc. and is not relevant to the choice of reflections for refinement. R-factors based on F^2^ are statistically about twice as large as those based on F, and R- factors based on ALL data will be even larger.

### Fractional atomic coordinates and isotropic or equivalent isotropic displacement parameters (Å^2^)

**Table:**

	*x*	*y*	*z*	*U*_iso_*/*U*_eq_	
S1A	0.62967 (4)	0.26781 (6)	0.97231 (3)	0.02670 (15)	
O1A	0.53829 (10)	0.50566 (16)	0.83979 (6)	0.0202 (3)	
O2A	0.65120 (10)	0.80602 (18)	0.99202 (6)	0.0279 (4)	
O3A	0.39540 (10)	0.75090 (15)	0.79654 (6)	0.0202 (3)	
N1A	0.57530 (11)	0.52837 (18)	0.95265 (7)	0.0171 (4)	
C1A	0.55815 (13)	0.6143 (2)	0.86534 (8)	0.0168 (5)	
C2A	0.59616 (14)	0.7378 (2)	0.85871 (9)	0.0174 (5)	
C3A	0.60336 (14)	0.7671 (2)	0.81616 (9)	0.0222 (5)	
H3AA	0.5827	0.7042	0.7860	0.027*	
C4A	0.64194 (15)	0.8918 (3)	0.81962 (10)	0.0272 (6)	
H4AA	0.6467	0.9158	0.7909	0.033*	
C5A	0.67402 (16)	0.9831 (3)	0.86471 (10)	0.0284 (6)	
H5AA	0.7002	1.0678	0.8659	0.034*	
C6A	0.66852 (15)	0.9527 (2)	0.90770 (10)	0.0253 (5)	
H6AA	0.6914	1.0141	0.9386	0.030*	
C7A	0.62811 (14)	0.8285 (2)	0.90376 (9)	0.0205 (5)	
C8A	0.61574 (14)	0.7684 (2)	0.94378 (9)	0.0195 (5)	
C9A	0.55364 (14)	0.6436 (2)	0.91497 (8)	0.0166 (4)	
C10A	0.45760 (14)	0.6739 (2)	0.89214 (8)	0.0167 (5)	
C11A	0.40732 (14)	0.7725 (2)	0.84090 (9)	0.0166 (4)	
C12A	0.37781 (13)	0.8917 (2)	0.85542 (8)	0.0155 (4)	
C13A	0.32921 (14)	1.0064 (2)	0.82197 (9)	0.0186 (5)	
H13A	0.3120	1.0158	0.7849	0.022*	
C14A	0.30687 (15)	1.1057 (2)	0.84438 (9)	0.0217 (5)	
H14A	0.2737	1.1843	0.8225	0.026*	
C15A	0.33283 (15)	1.0909 (2)	0.89928 (9)	0.0210 (5)	
H15A	0.3169	1.1599	0.9141	0.025*	
C16A	0.38156 (14)	0.9771 (2)	0.93249 (9)	0.0189 (5)	
H16A	0.3992	0.9682	0.9697	0.023*	
C17A	0.40395 (14)	0.8766 (2)	0.91014 (8)	0.0164 (4)	
C18A	0.45288 (14)	0.7428 (2)	0.93681 (9)	0.0175 (5)	
H18A	0.4212	0.6827	0.9456	0.021*	
H18C	0.5124	0.7611	0.9721	0.021*	
C19A	0.42138 (13)	0.5225 (2)	0.87689 (8)	0.0157 (4)	
H19A	0.4204	0.4941	0.8444	0.019*	
C20A	0.32991 (14)	0.4993 (2)	0.85811 (9)	0.0163 (4)	
C21A	0.30942 (14)	0.4529 (2)	0.89297 (9)	0.0189 (5)	
H21A	0.3547	0.4409	0.9323	0.023*	
C22A	0.22318 (15)	0.4240 (2)	0.87041 (9)	0.0219 (5)	
H22A	0.2105	0.3906	0.8945	0.026*	
C23A	0.15545 (15)	0.4430 (2)	0.81357 (9)	0.0227 (5)	
H23A	0.0968	0.4219	0.7986	0.027*	
C24A	0.17451 (15)	0.4935 (2)	0.77879 (10)	0.0252 (5)	
H24A	0.1286	0.5098	0.7399	0.030*	
C25A	0.26034 (14)	0.5199 (2)	0.80094 (9)	0.0200 (5)	
H25A	0.2725	0.5532	0.7766	0.024*	
C26A	0.49677 (14)	0.4401 (2)	0.92853 (9)	0.0183 (5)	
H26A	0.4853	0.4305	0.9567	0.022*	
C27A	0.51469 (14)	0.2975 (2)	0.91563 (10)	0.0239 (5)	
H27A	0.5030	0.2972	0.8785	0.029*	
H27C	0.4780	0.2259	0.9155	0.029*	
C28A	0.65485 (14)	0.4502 (2)	0.97413 (9)	0.0203 (5)	
H28A	0.7029	0.4790	1.0133	0.024*	
H28C	0.6734	0.4670	0.9503	0.024*	
S1B	1.12519 (4)	0.51070 (6)	0.97238 (3)	0.02524 (15)	
O1B	1.03573 (10)	0.27798 (16)	0.83636 (6)	0.0218 (3)	
O2B	1.12668 (10)	−0.03342 (16)	0.98297 (6)	0.0242 (4)	
O3B	0.88255 (10)	0.04463 (16)	0.78666 (6)	0.0211 (3)	
N1B	1.06431 (11)	0.25445 (18)	0.94627 (7)	0.0169 (4)	
C1B	1.05240 (13)	0.1689 (2)	0.86112 (9)	0.0173 (5)	
C2B	1.09053 (14)	0.0444 (2)	0.85552 (9)	0.0181 (5)	
C3B	1.10378 (14)	0.0155 (2)	0.81586 (9)	0.0221 (5)	
H3BA	1.0869	0.0796	0.7867	0.027*	
C4B	1.14246 (15)	−0.1098 (3)	0.82023 (10)	0.0266 (5)	
H4BA	1.1515	−0.1326	0.7933	0.032*	
C5B	1.16840 (16)	−0.2033 (3)	0.86366 (10)	0.0295 (6)	
H5BA	1.1950	−0.2884	0.8658	0.035*	
C6B	1.15601 (15)	−0.1742 (2)	0.90362 (10)	0.0241 (5)	
H6BA	1.1738	−0.2377	0.9331	0.029*	
C7B	1.11677 (14)	−0.0489 (2)	0.89915 (9)	0.0193 (5)	
C8B	1.09942 (14)	0.0095 (2)	0.93695 (9)	0.0191 (5)	
C9B	1.04226 (14)	0.1407 (2)	0.90805 (8)	0.0165 (4)	
C10B	0.94408 (14)	0.1170 (2)	0.88260 (8)	0.0166 (4)	
C11B	0.89434 (14)	0.0205 (2)	0.83098 (8)	0.0163 (4)	
C12B	0.86614 (14)	−0.1020 (2)	0.84504 (9)	0.0164 (4)	
C13B	0.82083 (14)	−0.2189 (2)	0.81213 (9)	0.0190 (5)	
H13B	0.8037	−0.2272	0.7750	0.023*	
C14B	0.80152 (15)	−0.3220 (2)	0.83486 (9)	0.0218 (5)	
H14B	0.7702	−0.4019	0.8131	0.026*	
C15B	0.82820 (15)	−0.3090 (2)	0.89015 (9)	0.0211 (5)	
H15B	0.8155	−0.3814	0.9056	0.025*	
C16B	0.87255 (14)	−0.1927 (2)	0.92241 (9)	0.0195 (5)	
H16B	0.8899	−0.1846	0.9596	0.023*	
C17B	0.89134 (13)	−0.0879 (2)	0.89954 (8)	0.0157 (4)	
C18B	0.93578 (14)	0.0482 (2)	0.92581 (9)	0.0177 (5)	
H18B	0.9942	0.0337	0.9624	0.021*	
H18D	0.9002	0.1064	0.9321	0.021*	
C19B	0.91199 (13)	0.2697 (2)	0.86880 (8)	0.0164 (4)	
H19B	0.9129	0.2994	0.8372	0.020*	
C20B	0.81980 (14)	0.2937 (2)	0.84906 (9)	0.0166 (4)	
C21B	0.79908 (15)	0.3294 (2)	0.88491 (9)	0.0200 (5)	
H21B	0.8443	0.3371	0.9244	0.024*	
C22B	0.71272 (15)	0.3535 (2)	0.86304 (10)	0.0250 (5)	
H22B	0.6997	0.3785	0.8878	0.030*	
C23B	0.64529 (15)	0.3417 (2)	0.80574 (10)	0.0263 (5)	
H23B	0.5865	0.3597	0.7910	0.032*	
C24B	0.66510 (15)	0.3031 (2)	0.77019 (10)	0.0260 (5)	
H24B	0.6193	0.2920	0.7309	0.031*	
C25B	0.75119 (14)	0.2807 (2)	0.79155 (9)	0.0202 (5)	
H25B	0.7637	0.2559	0.7665	0.024*	
C26B	0.98754 (13)	0.3470 (2)	0.92214 (9)	0.0180 (5)	
H26B	0.9743	0.3561	0.9494	0.022*	
C27B	1.01105 (14)	0.4886 (2)	0.91226 (9)	0.0228 (5)	
H27B	0.9744	0.5623	0.9112	0.027*	
H27D	1.0034	0.4900	0.8764	0.027*	
C28B	1.14705 (14)	0.3275 (2)	0.97163 (9)	0.0210 (5)	
H28D	1.1681	0.3122	0.9491	0.025*	
H28E	1.1923	0.2938	1.0105	0.025*	

### Atomic displacement parameters (Å^2^)

**Table:**

	*U*^11^	*U*^22^	*U*^33^	*U*^12^	*U*^13^	*U*^23^
S1A	0.0213 (3)	0.0209 (3)	0.0327 (3)	0.0081 (2)	0.0139 (3)	0.0062 (3)
O1A	0.0215 (8)	0.0199 (9)	0.0223 (8)	−0.0007 (7)	0.0150 (7)	−0.0036 (7)
O2A	0.0270 (9)	0.0345 (10)	0.0219 (8)	−0.0055 (8)	0.0148 (7)	−0.0085 (7)
O3A	0.0248 (9)	0.0214 (9)	0.0183 (7)	0.0026 (7)	0.0152 (7)	0.0017 (6)
N1A	0.0166 (10)	0.0172 (10)	0.0187 (9)	0.0026 (8)	0.0114 (8)	0.0021 (7)
C1A	0.0129 (11)	0.0209 (12)	0.0173 (10)	0.0054 (9)	0.0096 (9)	0.0026 (9)
C2A	0.0136 (11)	0.0193 (12)	0.0191 (10)	0.0021 (9)	0.0099 (9)	0.0006 (9)
C3A	0.0194 (12)	0.0271 (13)	0.0215 (11)	0.0014 (10)	0.0134 (10)	0.0000 (10)
C4A	0.0254 (13)	0.0310 (15)	0.0312 (13)	0.0008 (11)	0.0205 (11)	0.0059 (11)
C5A	0.0263 (14)	0.0220 (13)	0.0391 (14)	−0.0029 (11)	0.0212 (12)	0.0019 (11)
C6A	0.0215 (13)	0.0205 (13)	0.0315 (13)	−0.0014 (10)	0.0151 (11)	−0.0030 (10)
C7A	0.0155 (12)	0.0216 (12)	0.0219 (11)	0.0014 (9)	0.0102 (9)	−0.0019 (9)
C8A	0.0169 (11)	0.0215 (12)	0.0198 (11)	0.0020 (9)	0.0111 (9)	−0.0026 (9)
C9A	0.0165 (11)	0.0189 (11)	0.0161 (10)	0.0035 (9)	0.0109 (9)	0.0016 (9)
C10A	0.0187 (11)	0.0170 (11)	0.0179 (10)	0.0027 (9)	0.0129 (9)	0.0010 (9)
C11A	0.0159 (11)	0.0166 (11)	0.0193 (10)	−0.0013 (9)	0.0117 (9)	0.0013 (9)
C12A	0.0137 (11)	0.0146 (11)	0.0175 (10)	−0.0002 (8)	0.0093 (9)	0.0012 (8)
C13A	0.0204 (12)	0.0165 (11)	0.0186 (10)	0.0005 (9)	0.0119 (9)	0.0018 (9)
C14A	0.0246 (13)	0.0162 (12)	0.0211 (11)	0.0045 (10)	0.0125 (10)	0.0050 (9)
C15A	0.0249 (12)	0.0161 (12)	0.0241 (11)	0.0010 (10)	0.0161 (10)	−0.0011 (9)
C16A	0.0230 (12)	0.0175 (12)	0.0188 (10)	−0.0004 (9)	0.0141 (10)	−0.0006 (9)
C17A	0.0155 (11)	0.0158 (11)	0.0183 (10)	−0.0011 (9)	0.0105 (9)	0.0002 (9)
C18A	0.0201 (11)	0.0171 (11)	0.0189 (10)	0.0040 (9)	0.0138 (9)	0.0041 (9)
C19A	0.0172 (11)	0.0155 (11)	0.0168 (10)	0.0031 (9)	0.0116 (9)	0.0019 (8)
C20A	0.0171 (11)	0.0123 (11)	0.0218 (10)	0.0019 (9)	0.0132 (9)	0.0001 (9)
C21A	0.0206 (12)	0.0177 (12)	0.0190 (10)	0.0028 (9)	0.0125 (9)	0.0010 (9)
C22A	0.0277 (13)	0.0182 (12)	0.0303 (12)	0.0000 (10)	0.0232 (11)	0.0028 (10)
C23A	0.0177 (12)	0.0217 (13)	0.0295 (12)	−0.0024 (10)	0.0150 (10)	−0.0006 (10)
C24A	0.0200 (13)	0.0280 (14)	0.0232 (11)	0.0017 (10)	0.0110 (10)	0.0032 (10)
C25A	0.0220 (12)	0.0194 (12)	0.0197 (11)	0.0015 (9)	0.0133 (9)	0.0031 (9)
C26A	0.0184 (12)	0.0178 (12)	0.0202 (10)	0.0044 (9)	0.0125 (9)	0.0041 (9)
C27A	0.0201 (12)	0.0173 (12)	0.0304 (12)	0.0042 (10)	0.0135 (10)	0.0038 (10)
C28A	0.0165 (11)	0.0225 (12)	0.0214 (11)	0.0058 (9)	0.0113 (9)	0.0031 (9)
S1B	0.0198 (3)	0.0196 (3)	0.0297 (3)	−0.0061 (2)	0.0118 (3)	−0.0037 (2)
O1B	0.0233 (9)	0.0207 (9)	0.0262 (8)	0.0016 (7)	0.0177 (7)	0.0044 (7)
O2B	0.0241 (9)	0.0251 (9)	0.0193 (8)	0.0018 (7)	0.0112 (7)	0.0062 (7)
O3B	0.0240 (9)	0.0231 (9)	0.0179 (8)	−0.0024 (7)	0.0136 (7)	−0.0008 (6)
N1B	0.0149 (9)	0.0175 (10)	0.0179 (9)	−0.0032 (7)	0.0097 (8)	−0.0023 (7)
C1B	0.0128 (11)	0.0218 (12)	0.0168 (10)	−0.0021 (9)	0.0088 (9)	−0.0004 (9)
C2B	0.0136 (11)	0.0210 (12)	0.0169 (10)	−0.0014 (9)	0.0080 (9)	−0.0004 (9)
C3B	0.0190 (12)	0.0268 (13)	0.0229 (11)	−0.0003 (10)	0.0140 (10)	−0.0003 (10)
C4B	0.0263 (13)	0.0292 (14)	0.0283 (12)	−0.0008 (11)	0.0188 (11)	−0.0059 (11)
C5B	0.0301 (14)	0.0231 (13)	0.0373 (14)	0.0034 (11)	0.0217 (12)	−0.0024 (11)
C6B	0.0225 (13)	0.0191 (13)	0.0277 (12)	0.0017 (10)	0.0139 (10)	0.0015 (10)
C7B	0.0134 (11)	0.0198 (12)	0.0207 (11)	−0.0012 (9)	0.0085 (9)	0.0005 (9)
C8B	0.0153 (11)	0.0191 (12)	0.0186 (10)	−0.0047 (9)	0.0082 (9)	−0.0024 (9)
C9B	0.0159 (11)	0.0177 (11)	0.0175 (10)	−0.0013 (9)	0.0111 (9)	−0.0008 (9)
C10B	0.0175 (11)	0.0166 (11)	0.0178 (10)	−0.0041 (9)	0.0119 (9)	−0.0035 (9)
C11B	0.0160 (11)	0.0174 (11)	0.0176 (10)	0.0008 (9)	0.0115 (9)	−0.0006 (9)
C12B	0.0157 (11)	0.0143 (11)	0.0195 (10)	0.0013 (9)	0.0109 (9)	0.0009 (9)
C13B	0.0199 (12)	0.0179 (12)	0.0173 (10)	0.0000 (9)	0.0105 (9)	−0.0020 (9)
C14B	0.0249 (13)	0.0152 (12)	0.0235 (11)	−0.0041 (10)	0.0140 (10)	−0.0043 (9)
C15B	0.0255 (13)	0.0164 (12)	0.0230 (11)	−0.0028 (10)	0.0157 (10)	0.0022 (9)
C16B	0.0220 (12)	0.0179 (12)	0.0203 (11)	−0.0030 (9)	0.0138 (10)	−0.0021 (9)
C17B	0.0136 (11)	0.0145 (11)	0.0185 (10)	−0.0004 (8)	0.0097 (9)	−0.0011 (8)
C18B	0.0189 (12)	0.0195 (12)	0.0176 (10)	−0.0037 (9)	0.0125 (9)	−0.0018 (9)
C19B	0.0181 (11)	0.0143 (11)	0.0182 (10)	−0.0031 (9)	0.0119 (9)	−0.0028 (8)
C20B	0.0171 (11)	0.0118 (11)	0.0234 (11)	−0.0037 (9)	0.0136 (9)	−0.0019 (9)
C21B	0.0215 (12)	0.0183 (12)	0.0220 (11)	−0.0021 (9)	0.0143 (10)	−0.0037 (9)
C22B	0.0271 (14)	0.0217 (13)	0.0338 (13)	−0.0030 (10)	0.0225 (11)	−0.0080 (10)
C23B	0.0167 (12)	0.0243 (13)	0.0352 (13)	0.0001 (10)	0.0145 (11)	−0.0039 (11)
C24B	0.0212 (13)	0.0246 (13)	0.0251 (12)	−0.0021 (10)	0.0106 (10)	−0.0029 (10)
C25B	0.0208 (12)	0.0199 (12)	0.0218 (11)	−0.0008 (9)	0.0141 (10)	−0.0027 (9)
C26B	0.0158 (11)	0.0188 (12)	0.0203 (10)	−0.0018 (9)	0.0116 (9)	−0.0012 (9)
C27B	0.0180 (12)	0.0186 (12)	0.0276 (12)	−0.0033 (9)	0.0118 (10)	−0.0026 (10)
C28B	0.0186 (12)	0.0219 (13)	0.0229 (11)	−0.0033 (10)	0.0129 (10)	−0.0003 (9)

### Geometric parameters (Å, °)

**Table:**

S1A—C27A	1.811 (2)	S1B—C27B	1.813 (2)
S1A—C28A	1.818 (2)	S1B—C28B	1.822 (2)
O1A—C1A	1.214 (2)	O1B—C1B	1.212 (2)
O2A—C8A	1.215 (2)	O2B—C8B	1.209 (2)
O3A—C11A	1.220 (2)	O3B—C11B	1.225 (2)
N1A—C9A	1.452 (3)	N1B—C9B	1.449 (3)
N1A—C28A	1.461 (3)	N1B—C28B	1.463 (3)
N1A—C26A	1.485 (3)	N1B—C26B	1.486 (3)
C1A—C2A	1.476 (3)	C1B—C2B	1.472 (3)
C1A—C9A	1.568 (3)	C1B—C9B	1.559 (3)
C2A—C3A	1.394 (3)	C2B—C3B	1.389 (3)
C2A—C7A	1.396 (3)	C2B—C7B	1.402 (3)
C3A—C4A	1.386 (3)	C3B—C4B	1.385 (3)
C3A—H3AA	0.9500	C3B—H3BA	0.9500
C4A—C5A	1.400 (3)	C4B—C5B	1.400 (3)
C4A—H4AA	0.9500	C4B—H4BA	0.9500
C5A—C6A	1.388 (3)	C5B—C6B	1.387 (3)
C5A—H5AA	0.9500	C5B—H5BA	0.9500
C6A—C7A	1.396 (3)	C6B—C7B	1.389 (3)
C6A—H6AA	0.9500	C6B—H6BA	0.9500
C7A—C8A	1.478 (3)	C7B—C8B	1.477 (3)
C8A—C9A	1.539 (3)	C8B—C9B	1.550 (3)
C9A—C10A	1.563 (3)	C9B—C10B	1.569 (3)
C10A—C11A	1.542 (3)	C10B—C11B	1.537 (3)
C10A—C18A	1.547 (3)	C10B—C18B	1.549 (3)
C10A—C19A	1.563 (3)	C10B—C19B	1.554 (3)
C11A—C12A	1.464 (3)	C11B—C12B	1.468 (3)
C12A—C17A	1.396 (3)	C12B—C17B	1.396 (3)
C12A—C13A	1.400 (3)	C12B—C13B	1.398 (3)
C13A—C14A	1.382 (3)	C13B—C14B	1.380 (3)
C13A—H13A	0.9500	C13B—H13B	0.9500
C14A—C15A	1.402 (3)	C14B—C15B	1.407 (3)
C14A—H14A	0.9500	C14B—H14B	0.9500
C15A—C16A	1.391 (3)	C15B—C16B	1.383 (3)
C15A—H15A	0.9500	C15B—H15B	0.9500
C16A—C17A	1.390 (3)	C16B—C17B	1.390 (3)
C16A—H16A	0.9500	C16B—H16B	0.9500
C17A—C18A	1.508 (3)	C17B—C18B	1.501 (3)
C18A—H18A	0.9900	C18B—H18B	0.9900
C18A—H18C	0.9900	C18B—H18D	0.9900
C19A—C20A	1.508 (3)	C19B—C20B	1.512 (3)
C19A—C26A	1.544 (3)	C19B—C26B	1.543 (3)
C19A—H19A	1.0000	C19B—H19B	1.0000
C20A—C21A	1.399 (3)	C20B—C25B	1.394 (3)
C20A—C25A	1.399 (3)	C20B—C21B	1.400 (3)
C21A—C22A	1.390 (3)	C21B—C22B	1.389 (3)
C21A—H21A	0.9500	C21B—H21B	0.9500
C22A—C23A	1.384 (3)	C22B—C23B	1.384 (3)
C22A—H22A	0.9500	C22B—H22B	0.9500
C23A—C24A	1.389 (3)	C23B—C24B	1.386 (3)
C23A—H23A	0.9500	C23B—H23B	0.9500
C24A—C25A	1.381 (3)	C24B—C25B	1.386 (3)
C24A—H24A	0.9500	C24B—H24B	0.9500
C25A—H25A	0.9500	C25B—H25B	0.9500
C26A—C27A	1.526 (3)	C26B—C27B	1.526 (3)
C26A—H26A	1.0000	C26B—H26B	1.0000
C27A—H27A	0.9900	C27B—H27B	0.9900
C27A—H27C	0.9900	C27B—H27D	0.9900
C28A—H28A	0.9900	C28B—H28D	0.9900
C28A—H28C	0.9900	C28B—H28E	0.9900
			
C27A—S1A—C28A	91.53 (10)	C27B—S1B—C28B	91.36 (10)
C9A—N1A—C28A	117.51 (17)	C9B—N1B—C28B	118.27 (17)
C9A—N1A—C26A	109.83 (16)	C9B—N1B—C26B	110.40 (16)
C28A—N1A—C26A	113.82 (17)	C28B—N1B—C26B	114.26 (17)
O1A—C1A—C2A	127.13 (19)	O1B—C1B—C2B	127.08 (19)
O1A—C1A—C9A	125.02 (19)	O1B—C1B—C9B	124.77 (19)
C2A—C1A—C9A	107.72 (18)	C2B—C1B—C9B	108.03 (18)
C3A—C2A—C7A	121.6 (2)	C3B—C2B—C7B	121.2 (2)
C3A—C2A—C1A	128.6 (2)	C3B—C2B—C1B	129.0 (2)
C7A—C2A—C1A	109.73 (18)	C7B—C2B—C1B	109.78 (18)
C4A—C3A—C2A	117.3 (2)	C4B—C3B—C2B	117.8 (2)
C4A—C3A—H3AA	121.3	C4B—C3B—H3BA	121.1
C2A—C3A—H3AA	121.3	C2B—C3B—H3BA	121.1
C3A—C4A—C5A	121.2 (2)	C3B—C4B—C5B	121.1 (2)
C3A—C4A—H4AA	119.4	C3B—C4B—H4BA	119.5
C5A—C4A—H4AA	119.4	C5B—C4B—H4BA	119.5
C6A—C5A—C4A	121.5 (2)	C6B—C5B—C4B	121.3 (2)
C6A—C5A—H5AA	119.2	C6B—C5B—H5BA	119.4
C4A—C5A—H5AA	119.2	C4B—C5B—H5BA	119.4
C5A—C6A—C7A	117.4 (2)	C5B—C6B—C7B	117.8 (2)
C5A—C6A—H6AA	121.3	C5B—C6B—H6BA	121.1
C7A—C6A—H6AA	121.3	C7B—C6B—H6BA	121.1
C6A—C7A—C2A	120.9 (2)	C6B—C7B—C2B	120.9 (2)
C6A—C7A—C8A	129.0 (2)	C6B—C7B—C8B	128.7 (2)
C2A—C7A—C8A	110.06 (19)	C2B—C7B—C8B	110.34 (19)
O2A—C8A—C7A	126.4 (2)	O2B—C8B—C7B	126.8 (2)
O2A—C8A—C9A	125.4 (2)	O2B—C8B—C9B	125.5 (2)
C7A—C8A—C9A	108.11 (17)	C7B—C8B—C9B	107.63 (17)
N1A—C9A—C8A	114.17 (17)	N1B—C9B—C8B	114.46 (17)
N1A—C9A—C10A	100.53 (16)	N1B—C9B—C1B	116.27 (17)
C8A—C9A—C10A	114.04 (17)	C8B—C9B—C1B	101.87 (17)
N1A—C9A—C1A	116.30 (17)	N1B—C9B—C10B	100.19 (16)
C8A—C9A—C1A	101.25 (17)	C8B—C9B—C10B	112.82 (17)
C10A—C9A—C1A	111.14 (16)	C1B—C9B—C10B	111.76 (16)
C11A—C10A—C18A	105.14 (17)	C11B—C10B—C18B	105.07 (17)
C11A—C10A—C9A	113.93 (17)	C11B—C10B—C19B	113.95 (17)
C18A—C10A—C9A	112.73 (17)	C18B—C10B—C19B	113.60 (17)
C11A—C10A—C19A	112.94 (17)	C11B—C10B—C9B	113.69 (17)
C18A—C10A—C19A	113.69 (17)	C18B—C10B—C9B	111.76 (17)
C9A—C10A—C19A	98.71 (16)	C19B—C10B—C9B	99.06 (16)
O3A—C11A—C12A	127.63 (19)	O3B—C11B—C12B	127.59 (19)
O3A—C11A—C10A	124.24 (19)	O3B—C11B—C10B	124.19 (19)
C12A—C11A—C10A	108.13 (17)	C12B—C11B—C10B	108.22 (17)
C17A—C12A—C13A	121.60 (19)	C17B—C12B—C13B	121.5 (2)
C17A—C12A—C11A	109.95 (18)	C17B—C12B—C11B	109.41 (18)
C13A—C12A—C11A	128.44 (19)	C13B—C12B—C11B	129.05 (19)
C14A—C13A—C12A	118.22 (19)	C14B—C13B—C12B	118.40 (19)
C14A—C13A—H13A	120.9	C14B—C13B—H13B	120.8
C12A—C13A—H13A	120.9	C12B—C13B—H13B	120.8
C13A—C14A—C15A	120.4 (2)	C13B—C14B—C15B	120.1 (2)
C13A—C14A—H14A	119.8	C13B—C14B—H14B	119.9
C15A—C14A—H14A	119.8	C15B—C14B—H14B	119.9
C16A—C15A—C14A	121.2 (2)	C16B—C15B—C14B	121.2 (2)
C16A—C15A—H15A	119.4	C16B—C15B—H15B	119.4
C14A—C15A—H15A	119.4	C14B—C15B—H15B	119.4
C17A—C16A—C15A	118.72 (19)	C15B—C16B—C17B	118.9 (2)
C17A—C16A—H16A	120.6	C15B—C16B—H16B	120.5
C15A—C16A—H16A	120.6	C17B—C16B—H16B	120.5
C16A—C17A—C12A	119.87 (19)	C16B—C17B—C12B	119.8 (2)
C16A—C17A—C18A	128.43 (19)	C16B—C17B—C18B	128.11 (19)
C12A—C17A—C18A	111.67 (18)	C12B—C17B—C18B	112.11 (18)
C17A—C18A—C10A	105.08 (16)	C17B—C18B—C10B	104.84 (16)
C17A—C18A—H18A	110.7	C17B—C18B—H18B	110.8
C10A—C18A—H18A	110.7	C10B—C18B—H18B	110.8
C17A—C18A—H18C	110.7	C17B—C18B—H18D	110.8
C10A—C18A—H18C	110.7	C10B—C18B—H18D	110.8
H18A—C18A—H18C	108.8	H18B—C18B—H18D	108.9
C20A—C19A—C26A	117.91 (17)	C20B—C19B—C26B	118.09 (17)
C20A—C19A—C10A	117.38 (17)	C20B—C19B—C10B	115.59 (17)
C26A—C19A—C10A	102.11 (16)	C26B—C19B—C10B	102.28 (16)
C20A—C19A—H19A	106.2	C20B—C19B—H19B	106.7
C26A—C19A—H19A	106.2	C26B—C19B—H19B	106.7
C10A—C19A—H19A	106.2	C10B—C19B—H19B	106.7
C21A—C20A—C25A	117.4 (2)	C25B—C20B—C21B	117.9 (2)
C21A—C20A—C19A	124.95 (19)	C25B—C20B—C19B	118.00 (19)
C25A—C20A—C19A	117.56 (19)	C21B—C20B—C19B	124.09 (19)
C22A—C21A—C20A	120.3 (2)	C22B—C21B—C20B	120.4 (2)
C22A—C21A—H21A	119.8	C22B—C21B—H21B	119.8
C20A—C21A—H21A	119.8	C20B—C21B—H21B	119.8
C23A—C22A—C21A	121.3 (2)	C23B—C22B—C21B	121.1 (2)
C23A—C22A—H22A	119.3	C23B—C22B—H22B	119.4
C21A—C22A—H22A	119.3	C21B—C22B—H22B	119.4
C22A—C23A—C24A	119.0 (2)	C22B—C23B—C24B	118.8 (2)
C22A—C23A—H23A	120.5	C22B—C23B—H23B	120.6
C24A—C23A—H23A	120.5	C24B—C23B—H23B	120.6
C25A—C24A—C23A	119.8 (2)	C25B—C24B—C23B	120.4 (2)
C25A—C24A—H24A	120.1	C25B—C24B—H24B	119.8
C23A—C24A—H24A	120.1	C23B—C24B—H24B	119.8
C24A—C25A—C20A	122.1 (2)	C24B—C25B—C20B	121.3 (2)
C24A—C25A—H25A	118.9	C24B—C25B—H25B	119.3
C20A—C25A—H25A	118.9	C20B—C25B—H25B	119.3
N1A—C26A—C27A	108.50 (17)	N1B—C26B—C27B	107.79 (17)
N1A—C26A—C19A	104.34 (16)	N1B—C26B—C19B	104.02 (16)
C27A—C26A—C19A	115.54 (18)	C27B—C26B—C19B	115.93 (17)
N1A—C26A—H26A	109.4	N1B—C26B—H26B	109.6
C27A—C26A—H26A	109.4	C27B—C26B—H26B	109.6
C19A—C26A—H26A	109.4	C19B—C26B—H26B	109.6
C26A—C27A—S1A	104.12 (15)	C26B—C27B—S1B	103.79 (15)
C26A—C27A—H27A	110.9	C26B—C27B—H27B	111.0
S1A—C27A—H27A	110.9	S1B—C27B—H27B	111.0
C26A—C27A—H27C	110.9	C26B—C27B—H27D	111.0
S1A—C27A—H27C	110.9	S1B—C27B—H27D	111.0
H27A—C27A—H27C	109.0	H27B—C27B—H27D	109.0
N1A—C28A—S1A	107.26 (15)	N1B—C28B—S1B	106.62 (15)
N1A—C28A—H28A	110.3	N1B—C28B—H28D	110.4
S1A—C28A—H28A	110.3	S1B—C28B—H28D	110.4
N1A—C28A—H28C	110.3	N1B—C28B—H28E	110.4
S1A—C28A—H28C	110.3	S1B—C28B—H28E	110.4
H28A—C28A—H28C	108.5	H28D—C28B—H28E	108.6
			
O1A—C1A—C2A—C3A	12.4 (4)	O1B—C1B—C2B—C3B	−10.6 (4)
C9A—C1A—C2A—C3A	−171.6 (2)	C9B—C1B—C2B—C3B	173.2 (2)
O1A—C1A—C2A—C7A	−166.5 (2)	O1B—C1B—C2B—C7B	167.6 (2)
C9A—C1A—C2A—C7A	9.6 (2)	C9B—C1B—C2B—C7B	−8.6 (2)
C7A—C2A—C3A—C4A	−1.4 (3)	C7B—C2B—C3B—C4B	1.1 (3)
C1A—C2A—C3A—C4A	179.8 (2)	C1B—C2B—C3B—C4B	179.1 (2)
C2A—C3A—C4A—C5A	1.3 (3)	C2B—C3B—C4B—C5B	−0.8 (3)
C3A—C4A—C5A—C6A	0.0 (4)	C3B—C4B—C5B—C6B	0.3 (4)
C4A—C5A—C6A—C7A	−1.2 (3)	C4B—C5B—C6B—C7B	0.1 (4)
C5A—C6A—C7A—C2A	1.2 (3)	C5B—C6B—C7B—C2B	0.1 (3)
C5A—C6A—C7A—C8A	177.9 (2)	C5B—C6B—C7B—C8B	−177.3 (2)
C3A—C2A—C7A—C6A	0.1 (3)	C3B—C2B—C7B—C6B	−0.7 (3)
C1A—C2A—C7A—C6A	179.1 (2)	C1B—C2B—C7B—C6B	−179.1 (2)
C3A—C2A—C7A—C8A	−177.2 (2)	C3B—C2B—C7B—C8B	177.1 (2)
C1A—C2A—C7A—C8A	1.8 (2)	C1B—C2B—C7B—C8B	−1.3 (2)
C6A—C7A—C8A—O2A	−12.8 (4)	C6B—C7B—C8B—O2B	10.5 (4)
C2A—C7A—C8A—O2A	164.2 (2)	C2B—C7B—C8B—O2B	−167.1 (2)
C6A—C7A—C8A—C9A	170.3 (2)	C6B—C7B—C8B—C9B	−171.7 (2)
C2A—C7A—C8A—C9A	−12.7 (2)	C2B—C7B—C8B—C9B	10.7 (2)
C28A—N1A—C9A—C8A	−69.7 (2)	C28B—N1B—C9B—C8B	70.3 (2)
C26A—N1A—C9A—C8A	158.05 (17)	C26B—N1B—C9B—C8B	−155.47 (17)
C28A—N1A—C9A—C10A	167.74 (17)	C28B—N1B—C9B—C1B	−48.2 (2)
C26A—N1A—C9A—C10A	35.5 (2)	C26B—N1B—C9B—C1B	86.1 (2)
C28A—N1A—C9A—C1A	47.7 (3)	C28B—N1B—C9B—C10B	−168.74 (17)
C26A—N1A—C9A—C1A	−84.5 (2)	C26B—N1B—C9B—C10B	−34.5 (2)
O2A—C8A—C9A—N1A	−34.0 (3)	O2B—C8B—C9B—N1B	36.7 (3)
C7A—C8A—C9A—N1A	142.94 (18)	C7B—C8B—C9B—N1B	−141.11 (18)
O2A—C8A—C9A—C10A	80.8 (3)	O2B—C8B—C9B—C1B	163.1 (2)
C7A—C8A—C9A—C10A	−102.3 (2)	C7B—C8B—C9B—C1B	−14.8 (2)
O2A—C8A—C9A—C1A	−159.8 (2)	O2B—C8B—C9B—C10B	−77.0 (3)
C7A—C8A—C9A—C1A	17.2 (2)	C7B—C8B—C9B—C10B	105.17 (19)
O1A—C1A—C9A—N1A	35.7 (3)	O1B—C1B—C9B—N1B	−37.1 (3)
C2A—C1A—C9A—N1A	−140.44 (18)	C2B—C1B—C9B—N1B	139.27 (18)
O1A—C1A—C9A—C8A	160.0 (2)	O1B—C1B—C9B—C8B	−162.2 (2)
C2A—C1A—C9A—C8A	−16.1 (2)	C2B—C1B—C9B—C8B	14.1 (2)
O1A—C1A—C9A—C10A	−78.5 (3)	O1B—C1B—C9B—C10B	77.1 (3)
C2A—C1A—C9A—C10A	105.37 (19)	C2B—C1B—C9B—C10B	−106.56 (19)
N1A—C9A—C10A—C11A	−167.79 (17)	N1B—C9B—C10B—C11B	168.47 (17)
C8A—C9A—C10A—C11A	69.6 (2)	C8B—C9B—C10B—C11B	−69.4 (2)
C1A—C9A—C10A—C11A	−44.1 (2)	C1B—C9B—C10B—C11B	44.7 (2)
N1A—C9A—C10A—C18A	72.5 (2)	N1B—C9B—C10B—C18B	−72.8 (2)
C8A—C9A—C10A—C18A	−50.1 (2)	C8B—C9B—C10B—C18B	49.4 (2)
C1A—C9A—C10A—C18A	−163.79 (17)	C1B—C9B—C10B—C18B	163.45 (17)
N1A—C9A—C10A—C19A	−47.83 (18)	N1B—C9B—C10B—C19B	47.22 (18)
C8A—C9A—C10A—C19A	−170.43 (16)	C8B—C9B—C10B—C19B	169.36 (16)
C1A—C9A—C10A—C19A	75.89 (19)	C1B—C9B—C10B—C19B	−76.55 (19)
C18A—C10A—C11A—O3A	−178.3 (2)	C18B—C10B—C11B—O3B	176.0 (2)
C9A—C10A—C11A—O3A	57.7 (3)	C19B—C10B—C11B—O3B	51.0 (3)
C19A—C10A—C11A—O3A	−53.8 (3)	C9B—C10B—C11B—O3B	−61.5 (3)
C18A—C10A—C11A—C12A	1.5 (2)	C18B—C10B—C11B—C12B	−4.8 (2)
C9A—C10A—C11A—C12A	−122.43 (19)	C19B—C10B—C11B—C12B	−129.81 (19)
C19A—C10A—C11A—C12A	125.99 (18)	C9B—C10B—C11B—C12B	117.67 (19)
O3A—C11A—C12A—C17A	179.3 (2)	O3B—C11B—C12B—C17B	−179.0 (2)
C10A—C11A—C12A—C17A	−0.5 (2)	C10B—C11B—C12B—C17B	1.9 (2)
O3A—C11A—C12A—C13A	0.5 (4)	O3B—C11B—C12B—C13B	0.8 (4)
C10A—C11A—C12A—C13A	−179.4 (2)	C10B—C11B—C12B—C13B	−178.4 (2)
C17A—C12A—C13A—C14A	−0.3 (3)	C17B—C12B—C13B—C14B	−0.3 (3)
C11A—C12A—C13A—C14A	178.4 (2)	C11B—C12B—C13B—C14B	179.9 (2)
C12A—C13A—C14A—C15A	0.2 (3)	C12B—C13B—C14B—C15B	−0.8 (3)
C13A—C14A—C15A—C16A	0.1 (3)	C13B—C14B—C15B—C16B	1.2 (3)
C14A—C15A—C16A—C17A	−0.4 (3)	C14B—C15B—C16B—C17B	−0.5 (3)
C15A—C16A—C17A—C12A	0.3 (3)	C15B—C16B—C17B—C12B	−0.6 (3)
C15A—C16A—C17A—C18A	−177.5 (2)	C15B—C16B—C17B—C18B	177.9 (2)
C13A—C12A—C17A—C16A	0.1 (3)	C13B—C12B—C17B—C16B	1.1 (3)
C11A—C12A—C17A—C16A	−178.88 (19)	C11B—C12B—C17B—C16B	−179.18 (19)
C13A—C12A—C17A—C18A	178.18 (19)	C13B—C12B—C17B—C18B	−177.67 (19)
C11A—C12A—C17A—C18A	−0.8 (2)	C11B—C12B—C17B—C18B	2.1 (2)
C16A—C17A—C18A—C10A	179.6 (2)	C16B—C17B—C18B—C10B	176.3 (2)
C12A—C17A—C18A—C10A	1.7 (2)	C12B—C17B—C18B—C10B	−5.1 (2)
C11A—C10A—C18A—C17A	−1.9 (2)	C11B—C10B—C18B—C17B	5.8 (2)
C9A—C10A—C18A—C17A	122.81 (18)	C19B—C10B—C18B—C17B	131.00 (18)
C19A—C10A—C18A—C17A	−125.90 (18)	C9B—C10B—C18B—C17B	−117.94 (18)
C11A—C10A—C19A—C20A	−65.5 (2)	C11B—C10B—C19B—C20B	65.9 (2)
C18A—C10A—C19A—C20A	54.2 (2)	C18B—C10B—C19B—C20B	−54.4 (2)
C9A—C10A—C19A—C20A	173.76 (17)	C9B—C10B—C19B—C20B	−173.00 (16)
C11A—C10A—C19A—C26A	163.90 (17)	C11B—C10B—C19B—C26B	−164.37 (17)
C18A—C10A—C19A—C26A	−76.4 (2)	C18B—C10B—C19B—C26B	75.3 (2)
C9A—C10A—C19A—C26A	43.20 (18)	C9B—C10B—C19B—C26B	−43.31 (18)
C26A—C19A—C20A—C21A	25.0 (3)	C26B—C19B—C20B—C25B	151.2 (2)
C10A—C19A—C20A—C21A	−97.8 (2)	C10B—C19B—C20B—C25B	−87.3 (2)
C26A—C19A—C20A—C25A	−151.99 (19)	C26B—C19B—C20B—C21B	−28.3 (3)
C10A—C19A—C20A—C25A	85.2 (2)	C10B—C19B—C20B—C21B	93.2 (2)
C25A—C20A—C21A—C22A	2.3 (3)	C25B—C20B—C21B—C22B	−1.3 (3)
C19A—C20A—C21A—C22A	−174.7 (2)	C19B—C20B—C21B—C22B	178.1 (2)
C20A—C21A—C22A—C23A	−1.4 (3)	C20B—C21B—C22B—C23B	0.6 (3)
C21A—C22A—C23A—C24A	−0.7 (3)	C21B—C22B—C23B—C24B	0.9 (4)
C22A—C23A—C24A—C25A	1.9 (3)	C22B—C23B—C24B—C25B	−1.8 (4)
C23A—C24A—C25A—C20A	−0.9 (4)	C23B—C24B—C25B—C20B	1.1 (4)
C21A—C20A—C25A—C24A	−1.2 (3)	C21B—C20B—C25B—C24B	0.5 (3)
C19A—C20A—C25A—C24A	176.0 (2)	C19B—C20B—C25B—C24B	−179.0 (2)
C9A—N1A—C26A—C27A	115.77 (18)	C9B—N1B—C26B—C27B	−116.42 (18)
C28A—N1A—C26A—C27A	−18.3 (2)	C28B—N1B—C26B—C27B	19.8 (2)
C9A—N1A—C26A—C19A	−7.9 (2)	C9B—N1B—C26B—C19B	7.2 (2)
C28A—N1A—C26A—C19A	−142.03 (17)	C28B—N1B—C26B—C19B	143.38 (17)
C20A—C19A—C26A—N1A	−153.37 (17)	C20B—C19B—C26B—N1B	151.82 (18)
C10A—C19A—C26A—N1A	−23.1 (2)	C10B—C19B—C26B—N1B	23.7 (2)
C20A—C19A—C26A—C27A	87.6 (2)	C20B—C19B—C26B—C27B	−90.0 (2)
C10A—C19A—C26A—C27A	−142.16 (18)	C10B—C19B—C26B—C27B	141.83 (18)
N1A—C26A—C27A—S1A	36.06 (19)	N1B—C26B—C27B—S1B	−37.88 (19)
C19A—C26A—C27A—S1A	152.76 (16)	C19B—C26B—C27B—S1B	−153.91 (16)
C28A—S1A—C27A—C26A	−35.51 (16)	C28B—S1B—C27B—C26B	37.12 (16)
C9A—N1A—C28A—S1A	−138.76 (15)	C9B—N1B—C28B—S1B	140.55 (15)
C26A—N1A—C28A—S1A	−8.4 (2)	C26B—N1B—C28B—S1B	8.0 (2)
C27A—S1A—C28A—N1A	25.99 (16)	C27B—S1B—C28B—N1B	−26.63 (15)

### Hydrogen-bond geometry (Å, °)

**Table:**

*D*—H···*A*	*D*—H	H···*A*	*D*···*A*	*D*—H···*A*
C15A—H15A···O2A^i^	0.95	2.51	3.235 (3)	134
C16B—H16B···N1B^ii^	0.95	2.48	3.383 (3)	159
C18B—H18B···O2B	0.99	2.33	3.070 (4)	131
C18A—H18C···O2A	0.99	2.40	3.167 (4)	133
C19A—H19A···O1A	1.00	2.37	3.075 (4)	127
C19B—H19B···O1B	1.00	2.40	3.086 (4)	125
C23A—H23A···O1B^iii^	0.95	2.50	3.209 (4)	132
C23B—H23B···O1A	0.95	2.58	3.227 (4)	126

Symmetry codes: (i) −*x*+1, −*y*+2, −*z*+2; (ii) −*x*+2, −*y*, −*z*+2; (iii) *x*−1, *y*, *z*.
